# Functional Deletion/Insertion Promoter Variants in *SCARB1* Associated With Increased Susceptibility to Lipid Profile Abnormalities and Coronary Heart Disease

**DOI:** 10.3389/fcvm.2021.800873

**Published:** 2022-01-13

**Authors:** Senlin Hu, Dong Hu, Haoran Wei, Shi-yang Li, Dong Wang, Chen-ze Li, Jiangang Jiang, Daowen Wang, Guanglin Cui, Daowu Wang

**Affiliations:** ^1^Division of Cardiology, Department of Internal Medicine, Tongji Hospital, Tongji Medical College, Huazhong University of Science and Technology, Wuhan, China; ^2^Hubei Province Key Laboratory of Genetics and Molecular Mechanisms of Cardiological Disorders, Huazhong University of Science and Technology, Wuhan, China; ^3^State Key Laboratory of Reproductive Medicine, The Center for Clinical Reproductive Medicine and Department of Cardiology, The First Affiliated Hospital of Nanjing Medical University, Nanjing, China

**Keywords:** SCARB, lipids, genetics, variant, coronary heart disease

## Abstract

**Background:** Genetic variants in Scavenger receptor Class B Type 1 (*SCARB1*) influencing high-density lipoprotein cholesterol (HDL-C) and coronary heart disease (CHD) risk were identified by recent genome-wide association studies. Further study of potential functional variants in *SCARB1* may provide new ideas of the complicated relationship between HDL-C and CHD.

**Methods:** 2000 bp in *SCARB1* promoter region was re-sequenced in 168 participants with extremely high plasma HDL-C and 400 control subjects. Putative risk alleles were identified using bioinformatics analysis and reporter-gene assays. Two indel variants, rs144334493 and rs557348251, respectively, were genotyped in 5,002 CHD patients and 5,175 control subjects. The underlying mechanisms were investigated.

**Results:** Through resequencing, 27 genetic variants were identified. Results of genotyping in 5,002 CHD patients and 5,175 control subjects revealed that rs144334493 and rs557348251 were significantly associated with increased risk of CHD [odds ratio (OR): 1.28, 95% confidence interval (CI): 1.09 to 1.52, *p* = 0.003; OR: 2.65, 95% CI: 1.66–4.24, *p* = 4.4 × 10^−5^). Subsequent mechanism experiments demonstrated that rs144334493 deletion allele attenuated forkhead box A1 (FOXA1) binding to the promoter region of *SCARB1*, while FOXA1 overexpression reversely increased SR-BI expression.

**Conclusion:** Genetic variants in *SCARB1* promoter region significantly associated with the plasma lipid levels by affecting SR-BI expression and contribute to the susceptibility of CHD.

## Introduction

Dyslipidemia is one of the major risk factors of coronary heart disease (CHD) (1). An inverse relationship between high-density lipoprotein (HDL) cholesterol (HDL-C) and the risk of CHD was identified by population-based studies (1, 2). However, clinical trials with drugs, niacin, and cholesteryl ester transfer protein (CETP) inhibitors, which could raise HDL-C level, have produced disappointing results (3). The causal role of HDL-C in cardio-protection has been questioned by genetic and randomized studies (4, 5), which suggests that HDL components rather than cholesterol may account for the anti-atherothrombotic effects of this lipoprotein. Indeed, HDL subspecies related to HDL function might provide new information to the prediction of cardiovascular disease risk (6). Although previous literatures found that individuals may not be protected against CHD by higher HDL-C level, we proposed that further human genetic analysis might provide new insight into the complicated relationship between HDL and CHD.

Scavenger receptor class B member 1 (SR-BI) is the major receptor for HDL (7). Transgenic mice overexpressing SR-BI was protected against atherosclerosis, while SR-BI deficiency could increase atherosclerosis, ignoring the increased levels of HDL-C, likely resulted from decreased hepatic delivery as well as clearance of cholesterol (8–13). To date, increasing evidences have demonstrated that variants of *SCARB1* were associated with plasma lipid levels (14), carotid intimal medial thickness (15), insulin sensitivity (16), and risk of CHD (17). In 2011, Vergeer et al. identified a new missense variant in the coding-region of *SCARB1*, which resulted in increased HDL cholesterol level, reduced intensity of efflux of cholesterol from macrophages, impaired platelets function, and diminished adrenal function (18). Recently, a rare homozygous variant in a subject with high HDL-C was identified by resequencing of *SCARB1*, which could raise HDL-C level and increase risk of CHD (19). However, most of the current studies mainly focused on the coding variant, only a few publications studied the non-coding region. Through genotyping of 690 participants, Hsu et al. identified an 11-bp deletion variant in the promoter region of *SCARB1* which explained a significant proportion of difference in individual HDL-C levels (20). Even so, the genome-wide association studies (GWAS) identified loci only explained a small proportion of HDL-C variation, suggesting that additional pathways and targets may exist. Furthermore, new molecular interactions can be defined through bioinformatic approaches of large-scale RNA sequencing data and genotypic data and further identify biologically meaningful gene networks which could control the metabolism of HDL and occurrence of related disease end points.

Because the plasma HDL-C concentration is inversely associated with increased risk of CHD, and SR-BI is the key molecule of HDL-C metabolism, further study of the sequencing variant of this gene may provide new insight into the complex relationship between HDL-C and CHD. Based on our resequencing results, we identified two variants, rs144334493, which located in the FOXA1 binding site, and an 11-bp deletion variant rs557348251 in the promoter region of *SCARB1*. We hypothesized that these two variants could modify SR-BI expression and contribute to the genetic susceptibility of CHD.

## Materials and Methods

### Study Design and Eligibility

The procedure of data collection, inclusion criteria, sample recruitment, and definition of risk factors were provided in our previous report (21, 22). Clinical characteristics of the study sample are listed in Table 1. In general, resequencing was conducted in 168 Han Chinese subjects with an HDL cholesterol level over the 95th percentile (>2.0 mmol/L) and a control group of 400 subjects. These participants were recruited in Wuhan Tongji Hospital from individuals who underwent routine health examinations. The 5,175 healthy control participants were community-based Chinese Han individuals recruited in 2012 from the general population, with ages ranging from 27 to 86 years. The CHD cohort, including 5,002 patients, was enrolled simultaneously between May 2004 and October 2015 from hospitalized patients in the Tongji Hospital. Details of the selection criteria, biochemical and clinical characteristics of the participants were described in our previous report (21, 22). All participants provided written informed consent. Experiments were performed in adherence to the Declaration of Helsinki. This study was conducted with the permission of institutional ethics committees of the local hospital.

**Table 1:** Baseline characteristics of the study samples.

**Cohort**	**High HDL-C sequencing cohort cases (*n* = 168)**	**Control sequencing cohort cases (*n* = 400)**	**The general population**	**The CHD population**
			**Controls (*n* = 5,175)**	**Cases (*n* = 5,002)**
Age, yrs	60.6 ± 10.6	59.7 ± 10.1	56.59 ± 10.01	60.36 ± 10.34
Male, %	60.8	34.9	48.04	72.98
BMI, kg/m^2^	21.2 ± 2.1	22.7 ± 2.8	23.72 ± 3.38	23.72 ± 3.38
TC, mmol/L	4.58 ± 0.40	4.57 ± 0.91	4.56 ± 0.95	4.14 ± 1.04
HDL-C, mmol/L	2.19 ± 0.19	1.52 ± 0.31	1.33 ± 0.33	1.01 ± 0.26
LDL-C, mmol/L	1.86 ± 0.47	2.57 ± 0.76	2.58 ± 0.76	2.45 ± 0.85
TG, mmol/L	0.77 ± 0.24	1.18 ± 0.50	1.32 ± 0.77	1.53 ± 1.01
Blood pressure, mm Hg				
Systolic	137.9 ± 26.9	133.3 ± 23.3	135.90 ± 23.37	134.24 ± 20.67
Diastolic	83.0 ± 16.0	81.6 ± 11.8	80.28 ± 12.20	80.17 ± 13.02
Hypertension	91 (54.2)	177 (44.3)	1,577 (30.47)	3,006 (60.10)
Type 2 diabetes	0	1 (0.3)	319 (6.16)	1,087 (21.73)
Current/ex-smoker	0	0	1,284 (24.81)	2,391 (47.80)
Previous myocardial infarction	0	0	0	1,832 (36.63)
Heart failure	0	0	0	40 (0.80)
History of cerebrovascular disease	0	0	343 (6.63)	600 (12.00)
Coronary angiography				
0-vessel disease	—	—	—	44 (0.88)
1-vessel disease	—	—	—	1,493 (29.85)
2-vessel disease	—	—	—	1,608 (32.15)
3-vessel disease	—	—	—	1,837 (36.73)
Left main trunk disease	—	—	—	407 (8.14)
Right coronary artery stenosis	—	—	—	3,535 (70.67)

*Values are mean ± SD or n (%). BMI, body mass index; CHD, coronary heart disease; HDL-C, high-density lipoprotein cholesterol; LDL-C, low-density lipoprotein cholesterol; TC, total cholesterol; TG, triglycerides*.

### Genetic Variation Screening

Sequence data at the *SCARB1* promoter region was generated by DNA sequencing. Polymerase-chain-reaction (PCR) covering the promoter region (*SCARB1* consensus sequence NC_000012.11 GRCh37.p13) was performed followed by Fluorescent dye-terminator cycle. Analysis of the products were carried out using an Applied Biosystems 3130xl capillary sequencer (Applied Biosystems, Foster City, CA). Putative polymorphisms were identified applying the Chromas program (Technelysium Pty. Ltd., Helensvale, Queensland, Australia) and confirmed by two independent observers. The identified variants were verified by repeat sequencing. Primers used for resequencing are given in Supplementary Table 1.

### *In silico* Analyses

HaploRegv4.1 (http://archive.broadinstitute.org/mammals/haploreg/haploreg.php) was used to identify the linkage disequilibrium of the SNPs (23). GWAS loci of *SCARB1* were derived from GWAS Catalog (www.ebi.ac.uk/gwas/home) (24). Possible functional variants were looked up in the RegulomeDB (http://www.regulomedb.org/index) database (25). Transcription factors with potential allele-specific binding efficacy were predicted with JASPAR2016 (http://jaspar2016.genereg.net/) (26).

### Functional Analysis

Bioinformatic analysis shows that among the 12 common SNP with MAF > 0.01, 4 were annotated by RegulomeDB with a score less or equal to 3. Since rs181338950 was proved to be no function in a previous study (20), sequencing flanking the other 3 common variant were constructed into PGL3-Basic vector and resultant plasmids (PGL3-WT and PGL3-MU of each polymorphism respectively) were transfected into HepG2 cells, to determine effects of the variants by detecting fluorescence intensity according to the manufacturer's instruction. Primers used for vector construction are given in Supplementary Table 2.

### Luciferase Assay

HepG2 cells (1 × 10^6^ cells per well) were used and co-transfected with 0.8 μg of PGL3-WT or PGL3-MU plasmid and 50 ng of Renilla luciferase, combined with lipofectamine 2000 (Invitrogen, Carlsbad, CA, USA) following the manufacturer's instruction. After 48 h of transection, cells were washed and lysed using Passive Lysis Buffer (SIRIUS, Pforzheim, Germany). The luciferase activities were detected with a luminometer (SIRIUS, Pforzheim, Germany) and calculated relative to Renilla luciferase activity and compared with negative control in each group. Among the 4 SNPs, the relation of rs144334493 and 4 predicted transcription factors were further investigated. HepG2 cells (1 × 10^6^ cells per well) were used and co-transfected with 0.8 ug of PGL3-insert or PGL3-deletion reporter vector of rs144334493 and 50 ng of Renilla luciferase, with 250ng pcDNA3.1(+)-FOXA1, pcDNA3.1(+)-CLOCK, pcDNA3.1(+)-MYC, pcDNA3.1(+)-MLXIPL, respectively, pcDNA3.1(+) empty vector was used as negative control. Luciferase activities were measured as above mentioned. Each experiment was performed three times with six replicates. Primers used for vector construction are given in Supplementary Table 3.

### Genotyping and Capillary Electrophoresis

SNP genotyping was performed in this study using the TaqMan SNP Genotyping Assay (Applied Biosystems). Genomic DNA extracted from peripheral leukocytes was used as previously reported (22). Probe and primer sequences applied for the TaqMan 5'-nuclease assay were produced by the ABI Primer Expression 3.0 software and then synthesized by Shanghai GeneCore BioTechnologies Co., Ltd, China. ABI 7900 HT Fast Real-Time PCR System (Applied Biosystems) was used for the analysis of the samples following the undermentioned conditions: 10 min at 95°C (enzyme activation), and 40 cycles at 95°C for 15 s followed by 60°C for 1 min (annealing/extension). An endpoint read was performed to determine the allelic discrimination results after amplification. Details were provide in our previous report regarding the performance for amplification reactions as well as the quality of genotyping (22). The 11 bp deletion variant rs557348251 was genotyped using Capillary electrophoresis method. In brief, PCR products were generated using FAM labeled forward primer. After purification, products were separated by Capillary electrophoresis with an Applied Biosystems 3130xl capillary sequencer (Applied Biosystems, Foster City, CA). Applied Biosystems GeneMapper 4.0 was used to identify putative polymorphisms. The identified variants were then confirmed by repeat sequencing. Probes and primers used are given in Supplementary Table 4.

### Western Blotting for SR-BI

Cell transfection experiments were performed using lipofectamine 2000 (Invitrogen, Carlsbad, CA, USA) following the instruction manual. In brief, HepG2 cells grown in six-well plates were transfected with 2,000 ng pcDNA3.1(+)-FOXA1, pcDNA3.1(+)-CLOCK, pcDNA3.1(+)-MYC, pcDNA3.1(+)-MLXIPL, respectively, pcDNA3.1(+) empty vector was used as negative control. After 48 h of transfection, the harvested cells were homogenized with lysis solution (50 mM Tris-Cl, pH 8.0; 1% Nonidet P-40; 150 mM NaCl; 0.1% SDS; 0.02% sodium azide; 0.5% sodium deoxycholate; and 1 μg/ml aprotinin) containing protease inhibitors (2 μg/ml leupeptin, 2 μg/ml aprotinin, 100 μg/ml phenylmethylsulfonyl fluoride). After centrifuging at 12,000 g for 20 min at 4°C, supernatant was collected. Protein concentration was measured with the BCA protein assay reagent kit (Boster, China). Lysates were resolved using 10% SDS-polyacrylamide gel electrophoresis. Products were transferred to polyvinylidene difluoride (PVDF) membranes. Following blocking with 5% non-fat milk, protein blots were probed with SR-BI (Lot No: BST17584233, Boster, China) or FLAG (#14793, Cell Signaling) antibody (1:1,000 diluted) and incubated with a peroxidase-conjugated secondary antibody. GAPDH was used as internal reference. Enhanced chemiluminescence reagents (Pierce Chemical, Rockford, IL) were used to visualize the protein bands, which were then quantified by densitometry.

### Chromatin Immunoprecipitation Assay

ChIP assays were performed using the ChIP Assay Kit (P2078, ChIP Assay Kit, Beyotime, China) according to the instruction manual. In brief, HepG2 or HEK293 cells (1 × 10^6^) were treated with 1% formaldehyde for 10 min at 37°C. Then, cells were collected and lysed in SDS lysis buffer. A sonic Dismembrator 550 (Fisher Scientific, Pittsburgh, PA) was used for eight 30-s cycles of sonication of the cell lysates. The input was phenol/chloroform-extracted and then ethanol-precipitated as internal positive control. After diluted in ChIP dilution buffer, samples were pre-cleared with salmon sperm DNA. Then the samples either received 1 μg rabbit IgG (negative control) or anti-Flag antibody (#14793, Cell Signaling) and incubated overnight at 4°C. Salmon sperm/protein A agarose slurry were used for the precipitation of the Abs, which were then extensively washed. 0.2 M NaCl was used to reverse the protein-DNA cross-links in 65°C for 4 h. Samples were then phenol/chloroform-extracted and ethanol-precipitated followed by ChIP-qPCR with control primers and primers that amplified 205 bases surrounding the rs144334493 variant in both rabbit IgG treated (control group) or anti-Flag antibody treated (anti-Flag group) group. ChIP-qPCR was done using ABI 7900HT Fast Real-Time PCR System (Applied Biosystems) with following conditions: 10 min at 95°C (enzyme activation), and 40 cycles at 95°C for 15 s followed by 60°C for 1 min (annealing/extension). Relative expression was calculated according to the ΔΔCq method using the Ct values provided by the manufacturer and compared between control and anti-flag group. Primers used for CHIP assay are given in Supplementary Table 5.

### Electrophoretic Mobility-Shift Assay

Commercial EMSA kit was used for the Electrophoretic mobility-shift assay (GS009, Chemiluminescent EMSA Kit, Beyotime, China) in this study. In brief, nuclear protein isolated from HepG2 cells applying Nuclear-Cytosol Extraction Kit (Applygen Technologies Inc, Beijing, China) was quantified with the BCA Protein Assay Kit (Boster, China). The rs144334493 insertion allele and deletion allele labeled oligonucleotides were commercially synthesized (Sangon Biotech, Shanghai, China). Gel shift assays were conducted with the LightShift Chemilluminescent EMSA kit (GS009, Beyotime) following the manufacturer's instructions. For competition experiments, unlabeled rs144334493 insertion or deletion oligonucleotides in 100 fold molar excess were added followed by the addition of the biotinylated probes as well as anti-Flag antibodies (#14793, Cell Signaling) used in supershift assays. Three independent experiments were performed for the analysis. Sequence of probes and primers are given in Supplementary Table 6.

### Real-Time RT-PCR Analysis

To determine the association between genotype and *in vivo* SR-BI expression, we included 64 control subjects in Wuhan Tongji Hospital who underwent routine health examinations. Characteristics of the study samples are given in Supplementary Table 7. Peripheral blood leukocytes were used for the extraction of total RNA using TRIzol reagent (Invitrogen). One microgram of total RNA was used for reverse transcription with PrimeScript RT reagent Kit with gDNA Eraser (Perfect Real Time) (Takara, Code No. RR047A). Real time PCR assays with SYBR Green (Takara, Code No. RR820L) were performed for measurement of SR-BI expression. ACTB was used as internal reference. Reaction was performed in triplicate with the primers listed in Supplementary Table 8. SR-BI expression was determined using the ΔΔCq method and compared between study subjects with different rs144334493 genotypes.

### Statistical Analysis

SPSS 13.0 (SPSS Inc, Chicago, IL) for Windows (Microsoft Corp, Redmond, Wash) was used for the statistical analyses. Linkage disequilibrium (LD) was calculated with Haploview version 4.2. Chi-square tests were performed to assess deviations of genotype frequency from the Hardy-Weinberg assumption. Multivariate linear regression analysis was performed to test associations between the SNPs and lipid traits using the additive genetic model adjusted by conventional vascular risk factors. Quantitative variables were analyzed using the Student *t*-test. The CHD risk was compared between disease and control groups using unconditional logistic regression analysis based on different genetic model after adjustment of conventional risk factors.

All biostatistics calculations were carried out using Prism5 (GraphPad). Comparisons among multiple conditions were conducted with ANOVA followed by *post hoc t*-tests. Comparisons between two groups were calculated using unpaired *t*-tests. Data are expressed as mean ± SEM of n experiments. The probability values were 2-sided, and *p* < 0.05 was considered significant.

## Results

### DNA Resequencing and Effects of Polymorphisms on Vitro Activity of the *SCARB1*

In total, 27 genetic variants were identified in our resequencing cohort consisted of 168 participants with extremely high plasma HDL-C and 400 control subjects, of which 12 were common variants with Minor allele frequency >0.01, as shown in Table 2. However, none of the distribution of the identified variants were statistically significant between the two groups.

**Table 2:** Characteristics of *SCARB1* promoter variants identified by resequencing in Control/High HDL-C cases.

Gene position^*^	**dbSNP ID^†^**	**Gene region**	**Maj> Min^**‡**^**	**Control**	**High HDL-C cases**	***P*-value**
				**MAF**	**MAF**	
chr12:125350530	rs79134272	Promoter	G/A	0.315	0.349	0.119
chr12:125350495	c.-2229C>T	Promoter	C/T	0.001	0.000	0.836
chr12:125350454	rs144334493	Promoter	GCT/-	0.110	0.103	0.621
chr12:125350368	c.-2102T>G	Promoter	T/G	0.000	0.007	0.160
chr12:125350360	c.-2094C>A	Promoter	C/A	0.001	0.000	0.657
chr12:125350320	rs4569100	Promoter	C/T	0.255	0.253	0.858
chr12:125349968	rs12580521	Promoter	G/C	0.253	0.253	0.993
chr12:125349897	c.-1631G>A	Promoter	G/A	0.001	0.000	0.658
chr12:125349849	rs12322330	Promoter	C/T	0.315	0.349	0.113
chr12:125349638	rs58157328	Promoter	-/C	0.083	0.135	0.208
chr12:125349519	rs36226544	Promoter	C/T	0.255	0.254	0.976
chr12:125349494	c.-1228G>A	Promoter	G/A	0.000	0.007	0.156
chr12:125349480	rs36226283	Promoter	C/T	0.257	0.254	0.914
chr12:125349374	rs112822039	Promoter	A/G	0.003	0.000	0.531
chr12:125349360	c.-1094C>T	Promoter	C/T	0.003	0.000	0.531
chr12:125349194	rs4765182	Promoter	G/C	0.329	0.350	0.334
chr12:125349192	rs36226280	Promoter	G/C	0.001	0.000	0.658
chr12:125349188	c.-922G>T	Promoter	G/T	0.001	0.000	0.658
chr12:125349080	rs373875675	Promoter	G/A	0.001	0.000	0.656
chr12:125349014	c.-748T>G	Promoter	T/G	0.001	0.000	0.656
chr12:125348988	rs59358115	Promoter	C/T	0.122	0.110	0.647
chr12:125348890	c.-624G>C	Promoter	G/C	0.001	0.000	0.656
chr12:125348877	c.-611G>A	Promoter	G/A	0.003	0.000	0.528
chr12:125348828	c.-562G>A	Promoter	G/A	0.001	0.000	0.656
chr12:125348582	c.-316C>T	Promoter	C/T	0.003	0.000	0.657
chr12:125348548	rs181338950	Promoter	C/T	0.103	0.096	0.624
chr12:125348536	rs557348251	Promoter	TCCCCGCCCCG/-	0.025	0.048	0.169

*All variants had at least a 90% genotype call rate and were in Hardy-Weinberg equilibrium (P > 0.05)*.

* *Base pair position is based on NCBI GRCh37*.

† *Polymorphisms are numbered relative to transcription start site*.

‡ *With major allele given first followed by minor allele; MAF, Minor allele frequency*.

*P-value: significance of minor allele frequency differences determined by Chi-square tests, cases vs. controls*.

**Figure 1:**
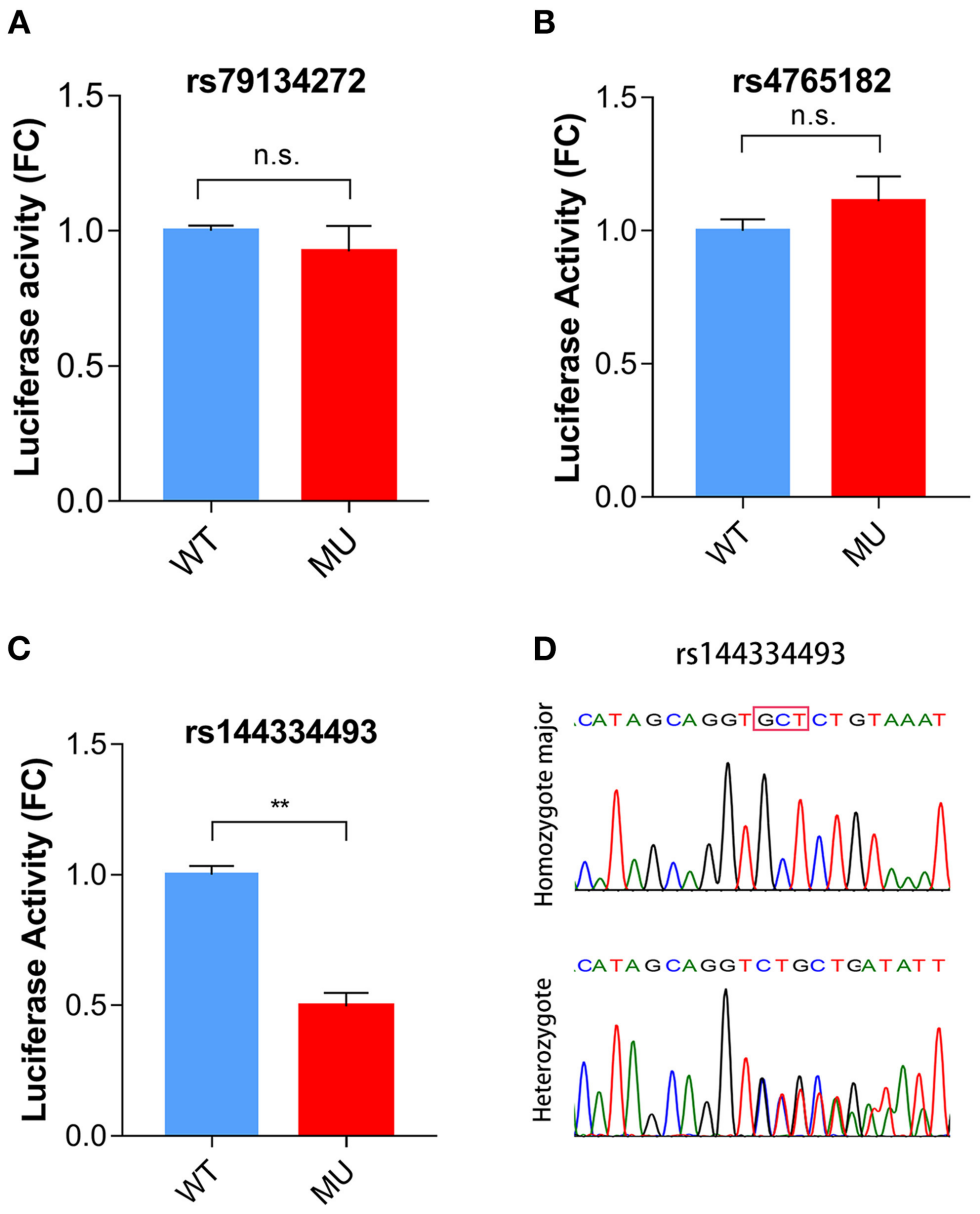
Functional analysis of the *SCARB1* promoter variants. Luciferase assay of the *SCARB1* promoter constructs carrying rs79134272 G/A **(A)**, rs4765182 G/C **(B)**, and rs144334493 GCT/ **(C)** variants, respectively, in HepG2 cells. *SCARB1* promoter activity is expressed as fold change of luciferase activity relative to PGL3-widetype. Luciferase activity was calculated relative to Renilla luciferase activity for each sample. Comparison was performed between mutant construct and the wild-type construct. Values are mean ± SE of three independent experiments. **(D)** Sequencing chromatogram for the rs144334493 insertion allele homozygote and heterozygote. FC, fold change; WT, wide type; MU, mutation type; n.s., no significant; **p < 0.01.

To identify potential functional variants, we defined the haploblock structure of the 12 common SNPs within the *SCARB1* promoter region using genotypes of the 400 control subjects (Supplementary Figure 1). Further bioinformatics analysis revealed that 4 of these common variants were annotated by RegulomeDB database with a score less or equal to 3 (Table 3), indicating that these variants are likely to be functional. Since rs181338950 was confirmed as a non-functional variant by a previous study through reporter gene assay (20), DNA sequences of 250 bp flanking the other three SNPs, respectively, were constructed into PGL3-Basic reporter vector. As shown in Figure 1, only rs144334493 variant had significant effect on the luciferase activity (Figures 1A–C). In HepG2 cells, reporter gene expression for rs144334493 mutant deletion allele was significantly decreased compared with rs144334493 wide-type insertion allele (50 ± 8.33%, *P* < 0.001) (Figures 1C,D). Furthermore, previous study identified a functional 11-basepair indel polymorphism rs557348251 which was also confirmed in our resequencing analysis without functional annotation (20). The rs557348251 polymorphism showed no linkage disequilibrium with the other 11 common SNPs and thus was also included in our following analysis.

**Table 3:** SNPs in strong linkage disequilibrium (r2>0.8) in the promoter region of *SCARB1* and RegulomeDB scores for predicted function.

**SNP**	**Chromosome**	**Position (GRCh37)**	**D^**′**^**	**r2**	**RegulomeDB score**
rs79134272	12	125350530	1	1	3a
rs12322330	12	125349849	0.975	0.951	5
rs4765182	12	125349194	0.974	0.888	2b
rs144334493	12	125350454	1	1	2b
rs59358115	12	125348988	0.972	0.852	4
rs181338950	12	125348548	0.97	0.866	2a
rs4569100	12	125350320	1	1	5
rs12580521	12	125349968	1	0.979	–
rs36226544	12	125349519	1	0.993	–
rs36226283	12	125349480	0.993	0.986	–
rs58157328	12	125349638	–	–	6
rs557348251	12	125348536	–	–	–

*RegulomeDB scores for predicted function (1 = likely to be functional; and 6 = not likely to be functional). Linkage disequilibrium values were obtained from the resequencing data. SNP indicates single nucleotide polymorphism*.

### Association of Rs144334493 and Rs557348251 Variant With Lipid Levels and Risk of CHD

**Figure 2:**
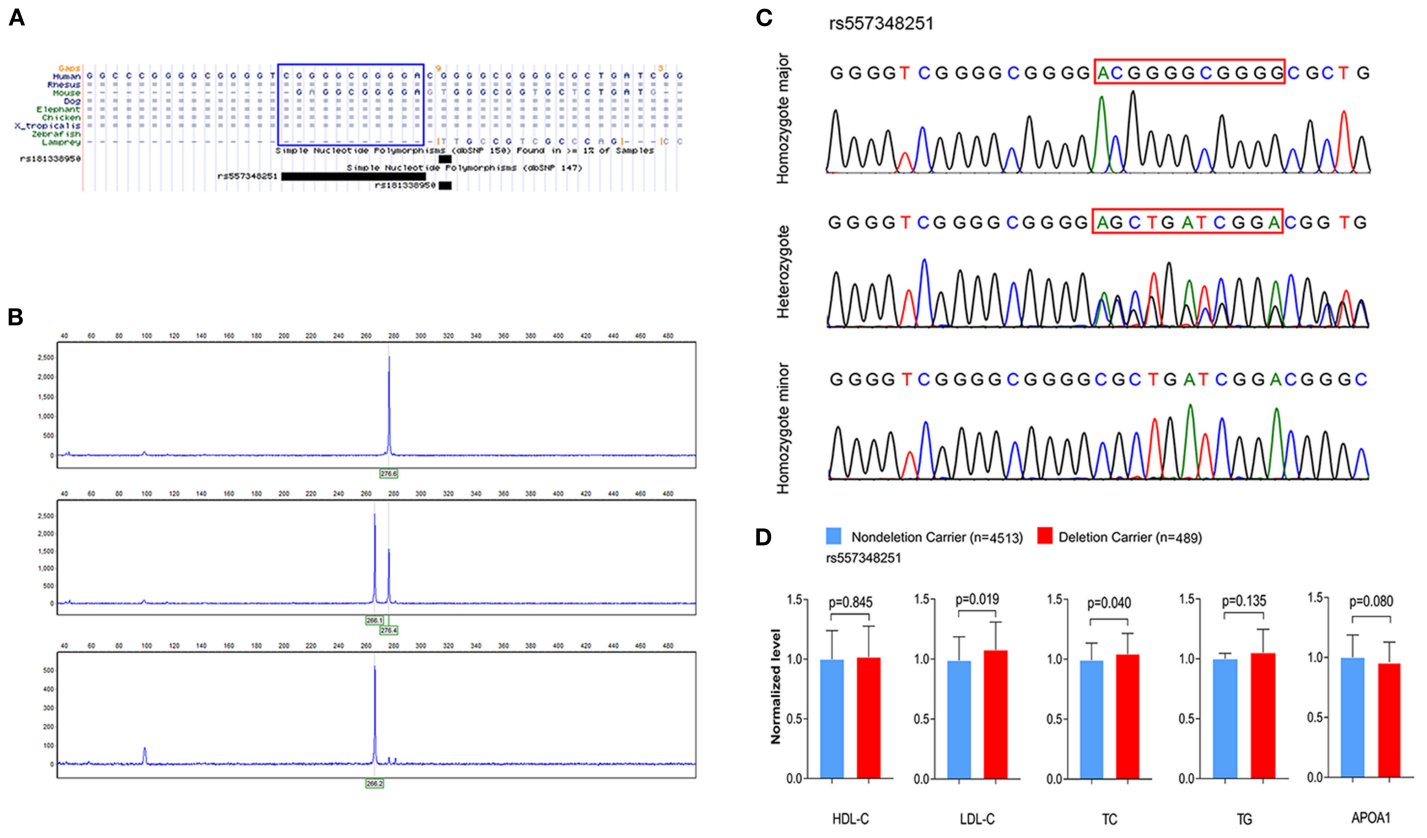
Genotyping and sequencing of rs557348251 and the association with plasma lipid levels. **(A)** Cross-species sequence alignment of *SCARB1* promoter region showing conservation of base pair positions in which rs557348251 variant was identified (included in blue Rectangle). **(B)** Capillary electrophoresis graphic of the three genotypes of rs557348251. Upper, homozygote major; middle, heterozygote; lower, homozygote minor. **(C)** Sequencing chromatogram for the three genotypes of rs557348251 (included in red Rectangle). **(D)** Association of rs557348251 genotype with lipid levels in the general population. Lipid levels are expressed as fold change relative to the non-deletion carrier group. HDL-C, high-density lipoprotein cholesterol; LDL-C, low-density lipoprotein cholesterol; TC, total cholesterol; TG, triglycerides.

To investigate the association between rs144334493, rs557348251 and plasma lipid levels and risk of CHD, we conducted genotyping using TaqMan probes and capillary electrophoresis in 5,175 control subjects and 5,002 CHD patients (Figures 2A–C). The relationship between rs144334493, rs557348251, and plasma lipid levels was analyzed in the 5,175 control subjects (Table 1). The rs144334493 polymorphism associated with decreased plasma levels of APOA1 (β = −0.188 mmol/L, *p* = 0.024) (Supplementary Table 9). Rs557348251 deletion allele was significantly associated with increased plasma TC and LDL-C levels (β = 0.270 mmol/L, *p* = 0.040; β = 0.293 mmol/L, *p* = 0.019, respectively) (Figure 2D), subjects carrying the rs557348251 deletion allele tend to have decreased plasma levels of APOA1 (β = −0.086 g/L, *p* = 0.080) (Figure 2D, Supplementary Table 10).

Results of the CHD patients showed that rs144334493 deletion allele was significantly associated with increased risk of CHD independent of conventional risk factors (additive model, OR: 1.28, 95% CI: 1.09–1.52, *p* = 0.003; dominant model, OR: 1.28, 95% CI: 1.06–1.54, *p* = 0.011; recessive model, OR: 2.01, 95%CI: 1.12–3.62, *p* = 0.020). Similar results were confirmed for rs557348251 in the case-control study (additive model, OR: 2.65, 95% CI: 1.66–4.24, *p* = 4.4^*^10^−5^; dominant model, OR: 3.00, 95% CI: 1.82–4.95, *p* = 1.8^*^10^−5^; recessive model, OR: 1.63, 95%CI = 0.27–9.88, *p* = 0.598) (Table 4).

**Table 4:** Association between rs144334493 variant with CHD.

**SNP rs ID**	**Function**	**Population**	**MAF**	***P*_alle_**	**MM**	**Mm**	**mm**	**Model**	**Crude ORs (95%CI)**	**Adjusted**	**Adjusted ORs (95%CI)**
**(M>m)**					***n*, (%)**	***n*, (%)**	***n*, (%)**			***P*_value_**	
rs144334493	Promoter	Control	0.127	0.003	3,948	1,140	87		1		1
(GCT>-)		CHD	0.131		3,784	1,124	94	Additive	1.05 (0.97–1.13)	0.003	1.28 (1.09–1.52)
								Dominant	1.04 (0.96–1.13)	0.011	1.28 (1.06–1.54)
								Recessive	1.18 (0.90–1.53)	0.020	2.01 (1.12–3.62)
rs557348251	Promoter	Control	0.024	1.16*10^−15^	4,933	234	8		1		1
(TCCCCGCCCCG>-)		CHD	0.052		4,513	454	35	Additive	2.66 (2.25–3.16)	4.4*10^−5^	2.65 (1.66–4.24)
								Dominant	2.86 (2.38-−3.42)	1.8*10^−5^	3.00 (1.82–4.95)
								Recessive	4.88 (2.25–10.55)	0.598	1.63 (0.27–9.88)

*CHD, Coronary Heart Disease; M, major allele; m, minor allele; MAF, minor allele frequency. P allele value and Crude odds ratio (OR) (95% confidence interval) were determined by a 95% two-sided χ 2 test, CHD vs. controls. Adjusted ORs (95% CI) and adjusted P-value were obtained with multivariate unconditional logistic regression analysis by adjusting for sex, age, smoking, hypertension, hyperlipidemia, diabetes, and body mass index*.

Combined analysis of the rs144334493 deletion allele and rs557348251 deletion allele with CHD was conducted. Results showed that carriers of both rs144334493 and rs557348251 deletion allele have significantly increased risk of CHD compared with the none deletion allele carriers, after adjusting for the conventional risk factors (OR: 3.88, 95%CI = 2.04–7.39, *p* = 3.8^*^10^−5^) (Table 5).

**Table 5:** Association between numbers of variations with CHD.

**Population**	**Num**.	**None**	**One**	**Two**	**Num**.	**Crude ORs (95%CI)**	**Adjusted**	**Adjusted ORs (95%CI)**
		***n*, (%)**	***n*, (%)**	***n*, (%)**			***P*_value_**	
Control		3,734	1,413	28				
CHD		3,398	1,501	103	None	1		1
					One	1.17 (1.07–1.27)	0.186	1.11 (0.95–1.29)
					Two	4.04 (2.66–6.15)	3.8*10^−5^	3.88 (2.04–7.39)

*CHD, Coronary Heart Disease; Num., numbers of the two deletion allele; None, none deletion allele carrier; One, carrier of rs144334493 or rs557348251 deletion allele; Two, carrier of both rs144334493 or rs557348251 deletion allele; Crude odds ratio (OR) [95% confidence interval (CI)] were determined by a 95% two-sided χ^2^ test, CHD vs. controls. Adjusted ORs (95% CI) and adjusted P-value were obtained with multivariate unconditional logistic regression analysis by adjusting for sex, age, smoking, hypertension, hyperlipidemia, diabetes, and body mass index*.

### Population Angiographic Characteristics of the CHD Cases

**Figure 3:**
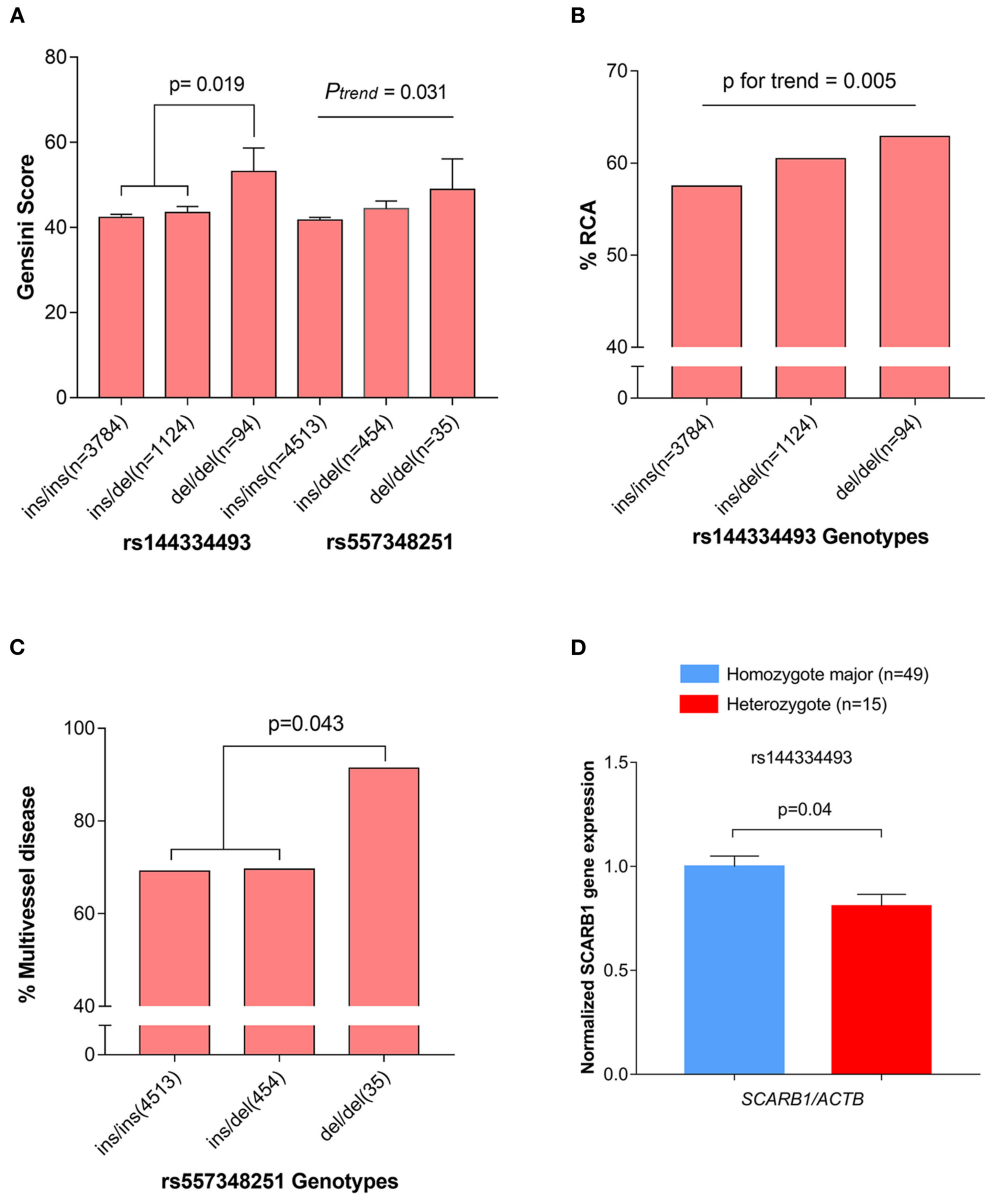
Association of rs144334493 and rs557348251 genotype with CHD gensini score, RCA stenosis, and multivissel disease of CHD. **(A)** The rs144334493 and rs557348251 variants associated with Gensini score adjusted by age, sex, hypertension, diabetes, and body mass index. **(B)** Proportion of CHD patients with RCA stenosis in different rs144334493 genotype. **(C)** Multivissel disease of CHD associated with variant rs557348251. **(D)** Association between variant rs144334493 and the *SCARB1* gene expression. Data are means ± SE from 64 independent samples, as described in the Methods, analyzed in three replicates normalized with ACTB mRNA. CHD, coronary heart disease; MAF, minor allele frequency; RCA, right coronary artery.

The CHD cases were documented angiographically as having >50% diameter stenosis in at least 1 coronary artery. The angiographic characteristics between different genotypes of rs144334493 and rs557348251 were analyzed (Supplementary Tables 11, 12). Both rs144334493 and rs557348251 deletion allele significantly associated with increased modified Gensini score (Figure 3A). Rs144334493 deletion allele carrier showed significantly increased proportion of incidence of right coronary artery stenosis and multi-vessel disease (*p* = 0.005, *p* = 0.009, respectively; Figure 3B). The rs557348251 homozygotes also showed increased proportion of multi-vessel disease (*p* = 0.043) (Figure 3C). Details of the association of the variants with angiographic characteristics were shown in Supplementary Tables 11, 12. Further *in vivo* SR-BI expression study revealed that rs144334493 variant was associated with decreased expression of SR-BI (Figure 3D).

### Variant Rs144334493 Located in the FOXA1 Binding Site

Subsequent bioinformatics analysis showed that rs144334493 occurred in the conservative binding site of transcription factor FOXA1, CLOCK, MYC, and MLXIPL. To test the prediction model that these transcription factors could functionally interact with rs144334493 sequence, luciferase reporter vectors containing the rs144334493 insert or deletion allele were co-transfected with either pcDNA3.1(+)-FOXA1, pcDNA3.1(+)-CLOCK pcDNA3.1(+)-MYC, pcDNA3.1(+)-MLXIPL constructs or pcDNA3.1(+) empty vector in HepG2 cells. The PGL3 vector containing the rs144334493 GCT insertion allele showed a significant increase in luciferase activity in the presence of FOXA1, while overexpression of MYC, MLXIPL, and CLOCK didn't influence the reporter activity (Supplementary Figure 2). It's worth noting that, in the presence of FOXA1, rs144334493 deletion allele construct still showed reduced luciferase activity than that of the rs144334493 insertion allele construct (18 ± 12%, *P* = 0.03) (Supplementary Figure 2).

**Figure 4:**
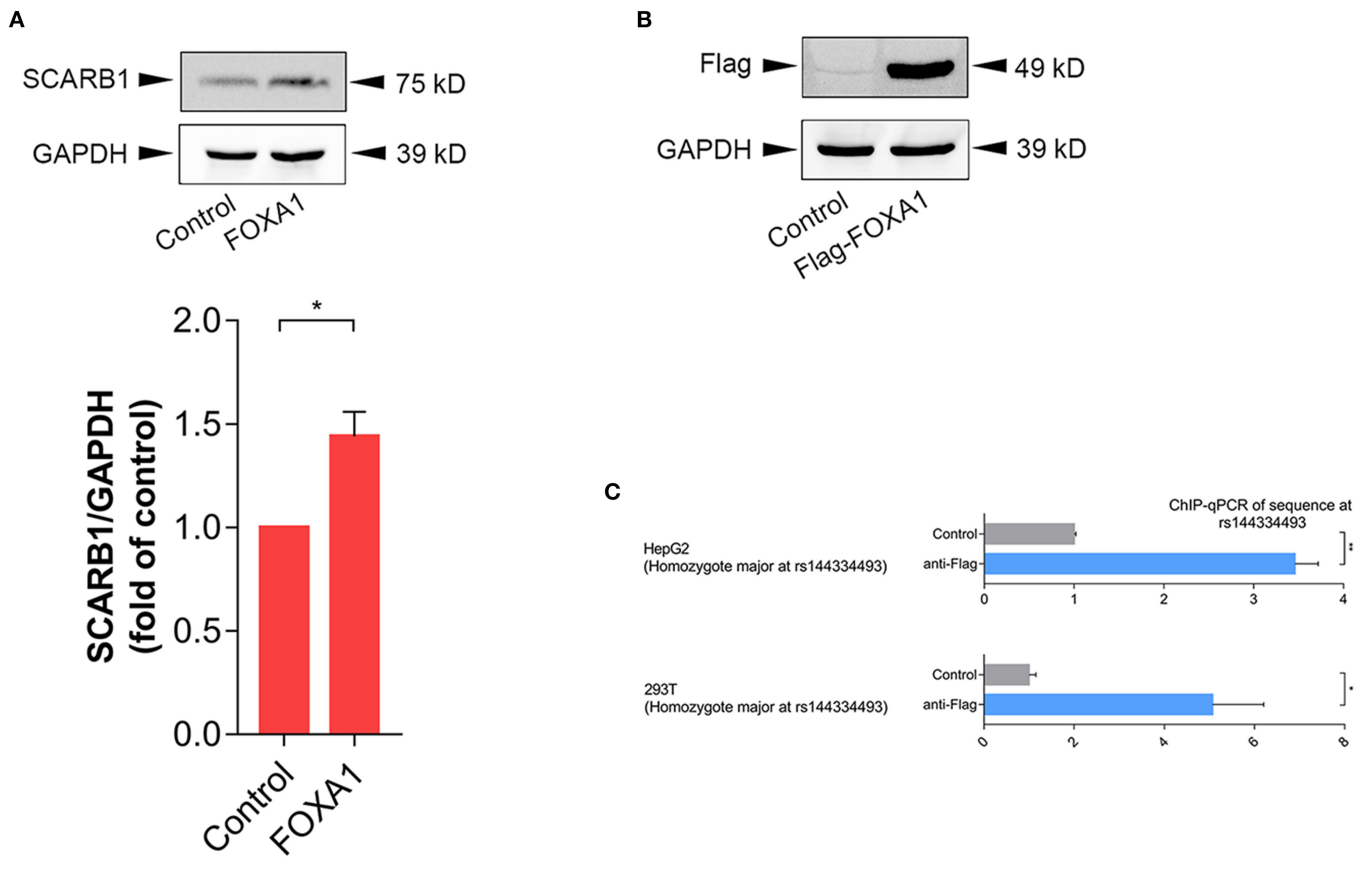
Rs144334493 located in the Forkhead Box A1 binding site. **(A)** Western blot showing FOXA1-induced expression of SR-BI in HepG2 cells. Columns = mean of 3 independent experiments, bars = SE. **(B)** Expression of Flag-tagged forkhead box A1 (FOXA1) in HepG2 cells. Flag-tagged FOXA1 construct was transfected into HepG2 cells, pcDNA3.1(+) empty vector was used as negative control. **(C)** CHIP assay in HepG2 and 293T cells showing *in vivo* interaction between FOXA1 and the rs144334493 sequence. Data are expressed as fold change relative to the control group. GADPH, glyceraldehyde 3-phosphate dehydrogenase; CHIP, Chromatin Immunoprecipitation; qPCR, quantitative polymerase chain reaction; **p < 0.01; *p < 0.05.

In order to further confirm the effect of FOXA1 on endogenous SR-BI expression, we sequenced six of the human hepatoma cell lines (including 97L, 7702, 7721, Hep1, Hep3B, and HepG2), all of which were identified to be rs144334493 insertion allele homozygotes (Supplementary Figure 3), and next we used HepG2 cell line to do experiments. Results showed that overexpression of FOXA1 significantly increased the expression of SR-BI, which was consistent with results of the reporter gene assay (Figure 4A). In contrast, the transcription factor CLOCK slightly decreased the expression of SR-BI, while both MYC and MLXIPL didn't influence the expression of SR-BI (Supplementary Figure 4). Chromatin immunoprecipitation experiment demonstrated that rs144334493 was located in the FOXA1 binding site in both HepG2 and 293T cells (Figures 4B,C). Further *in vitro* electrophoretic mobility shift assay revealed that FOXA1 may preferentially bind to the rs144334493 insertion allele rather than the deletion allele of rs144334493,although no supershift band was presented (Supplementary Figure 5). These results indicated that rs144334493 may affect SR-BI expression by allele-specific FOXA1 binding.

These results suggested that decreased SR-BI expression caused by genetic variations may be a possible pathogenic mechanism of CHD.

## Discussion

**Figure 5:**
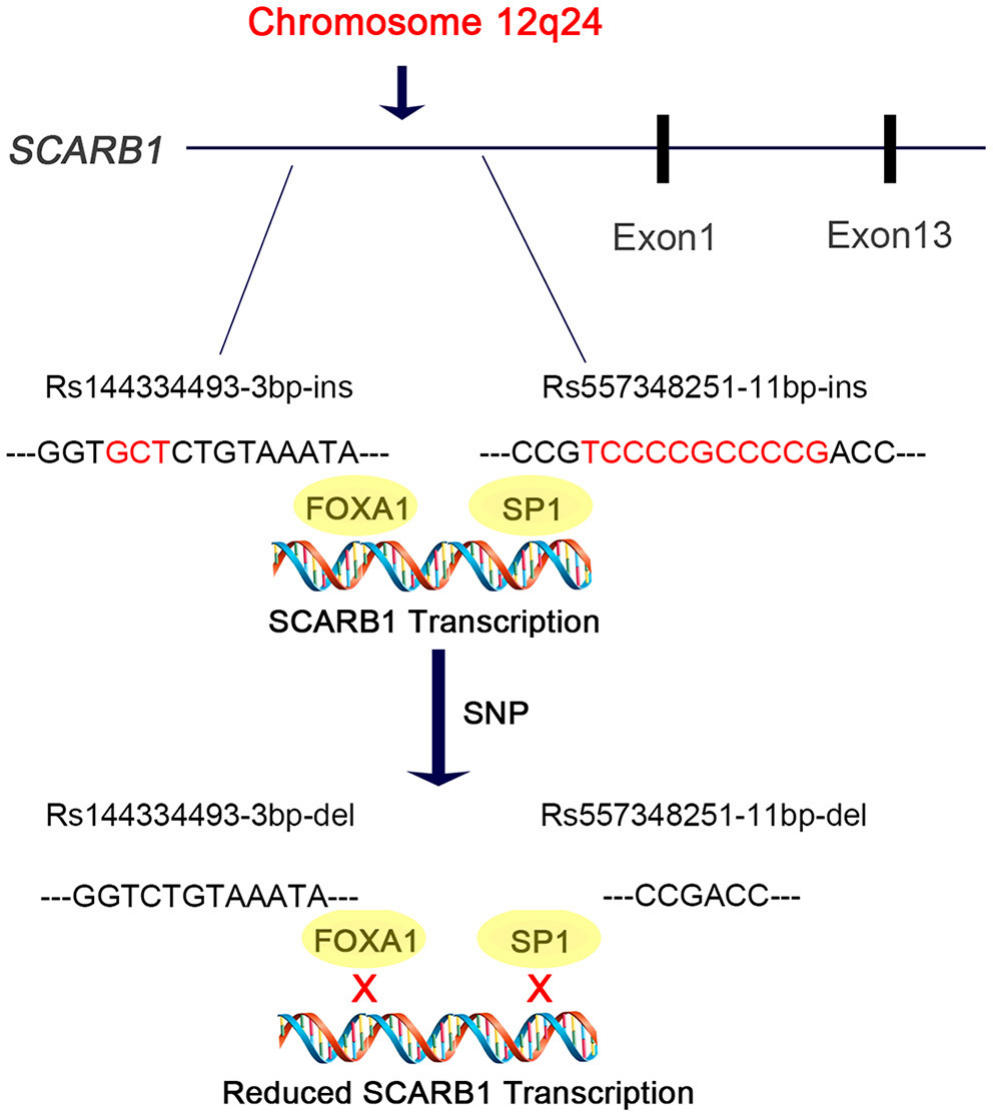
Diagram of the promoter sequence of the *SCARB1* gene. rs144334493 and rs557348251 were located in the promoter region of *SCARB1*. rs144334493 deletion allele was associated with loss of transcription factor FOXA1 binding site, while rs557348251 deletion allele was reported to influence SP1 binding. Both deletion alleles were associated with increased risk of CHD.

In this study, we discovered are that the deletion allele of the variants rs144334493 and rs557348251, in *SCARB1* promoter region, are significantly associated with increased risk of CHD (Figure 5). The rs557348251 variant dramatically increases risk of CHD in great amplitude and changes lipid levels. The deletion allele of rs144334493 attenuated FOXA1-induced expression of SR-BI. These associations are independent of conventional risk factors.

HDL-C level is inversely associated with CHD risk. *SCARB1* gene encoded the major receptor of HDL (7) which mediate the selective uptake of HDL-C. The normal function of this receptor is to transport cholesterol from the plasma into liver and steroidogenic organs like adrenal gland. *Scarb1* knockout in mice resulted in higher HDL-C and increased risk of atherosclerosis (11, 12, 27–30). Reversely, overexpression of *Scarb1* is associated with lower plasma levels of HDL-C and attenuated progression of atherosclerosis (8–10, 31). Therefore, targeting SR-BI is an attractive way to modulate plasma lipid level and hence reduce residual cardiovascular risk. With the development of the “Next-generation” sequencing technology, loss-of-function mutations of the *SCARB1* gene were identified to be associated with increased HDL-C levels and increased risk of ischemic vascular disease (18). This led to the hypothesis that adverse effect on CHD could be observed in carriers of *SCARB1* loss-of-function mutations. However, most of studies were focused on the coding region. In our study, *SCARB1* promoter region were re-sequenced in 400 control subjects and 168 participants with extremely high plasma HDL-C levels.

Up to now, GWASs were extensively used for the identification of common variants, termed SNPs, which were associated with different human traits. Nevertheless, protein-coding genes constitute only a limited proportion of the human genome. Study of the common variants in the non-coding regulatory region can provide new underlying mechanisms for the understanding of human traits and disease. Genetic studies have demonstrated that variant of *SCARB1* were associated with gene expression and plasma lipid levels (32), body mass index in white population (33), carotid intimal medial thickness (15), post-prandial lipid levels (16, 34), insulin sensitivity (16), risk of coronary heart disease (17), risk of peripheral arterial disease (35), age-related macular degeneration (36), and response to fenofibrate intervention (37). But study on the association between polymorphisms and risk of CHD in the promoter region of *SCARB1* is lacking (20). The SNP rs144334493 which is located in the promoter region of *SCARB1* was located in the DNaseI hypersensitivity site as annotated by ENCODE (http://genome.ucsc.edu/). No previously association of this variant with lipid and CHD risk was reported. Meanwhile, the rs557348251 variant was located in about 20 bp upstream of *SCARB1* transcription start site with a very high GC content. We found that rs144334493 deletion allele significantly influence relative luciferase expression. Thus, this lead to the hypothesis that rs144334493 may be a functional variant.

SR-BI is a key receptor of HDL-C metabolism and plays an important role in reverse cholesterol transport. Elevated HDL-C levels are inversely associated with cardiovascular disease. However, genetic variants which result in loss of function and attenuated expression and function of SR-BI are associated with increased risk of coronary heart disease (19). Recently, Lu et al. reported East Asian-specific coding variants influences lipid levels and contributes to coronary artery disease (38). However, none of these two indel variants in the promoter region of *SCARB1* was covered. In 2003, Hsu et al. reported an 11 bp deletion variant in the promoter region of *SCARB1* which can affect transcription factor SP1 binding and significantly influence HDL-C level while having no effect on other lipid components like LDL-C, TC, TG [(20), Figure 5]. Considering its limited sample size and sequencing coverage, whether there exit functional variant in the promoter region of *SCARB1* which may have both influence on lipid level and CHD risk is worth of research.

In this study, we identified two common functional variant rs144334493 and rs557348251 in the promoter region of *SCARB1* which were significantly related to the risk of CHD. The 11 bp indel variant rs557348251 was also associated with increased LDL-C. According to our resequencing results, rs59358115 and rs181338950, were in strong linkage disequilibrium (LD) (*r*^2^ > 0.8) with rs144334493, respectively. Furthermore, based on the 1,000 Genome data (http://grch37.ensembl.org/Homo_sapiens/), rs59358115 and rs181338950 were in modest LD (*r*^2^=0.013, D′ = 1) with the known GWAS loci rs11057864 (Supplementary Table 13). Indicating that rs144334493 may be the causal functional variant. Functional investigation demonstrated that the rs144334493 variant was located in the conserve binding site of transcription factor FOXA1, which can promote the expression of SR-BI, as confirmed by western blot assay. FOXA1 encodes a member of the forkhead class of DNA-binding proteins, and can activate liver-specific transcripts such as albumin (39). It also participants in the development of breast cancer (40). However, whether FOXA1 participates in the regulation of lipid metabolism, especially on SR-BI function have not been investigated. In this study, we firstly discovered that FOXA1 can increase the expression of SR-BI through conserved binding site flanking rs144334493. The association of the rs557348251 variant with CHD risk was more robustly than that of rs144334493, accompany with only a modest increase of plasma TC and LDL levels without significantly changes in HDL level. This discrepancy may indicate the complex relationship between HDL and CHD. Actually, through genotyping effort, Helgadottir et al. recently found that rare *SCARB1* mutations associate with high-density lipoprotein cholesterol but not with coronary artery disease (14). Large-scale studies are required to illuminate the association of SR-BI, HDL-C, and CHD.

There are some limitations of our study. Firstly, we cannot rule out the exist of other neighboring genes or regions in strong linkage disequilibrium with the identified variants which might explain the significant association with CHD. Secondly, the CHD study was conducted in patients who undergoing PCI and no replication study was conducted. Further larger patient populations are needed to confirm our findings. Thirdly, *in vitro* EMSA assay indicated allele-specific binding of FOXA1 at the rs144334493 site, however, no significant supershift band was detected. The regulatory pattern of FOXA1 on SR-BI expression needs to be clarified in further studies. Another limitation of our study is that the relationship between plasma lipid levels, *SCARB1* gene variation and CHD development has not been well-established. Zanoni et al. demonstrated that loss-of function variant of *SCARB1* was associated with elevated level of subfractions of HDL and risk of CHD, which was associated with only a modest increase of HDL-C [Beta (SE) in SD = +0.57 (0.071)] (19). This result requires further elucidation in the future.

In conclusion, our findings provide evidence that the rs144334493 deletion allele in the promoter region of *SCARB1* might increase the risk of CHD, probably by attenuating FOXA1 binding, which would lead to the under-expression of SR-BI, and further cause the reduction of reverse cholesterol efflux and increased risk of CHD. Meanwhile, another 11 bp deletion promoter variant rs557348251 also significantly associated with increased plasma levels of TC and LDL-C accompanied by strongly increased susceptibility to CHD. These results might help to improve the understanding of HDL-C metabolism, the CHD genetic risk evaluation and further prevention or therapy strategies of CHD by targeting SR-BI.

## Data Availability Statement

The datasets presented in this study can be found in online repositories. The names of the repository/repositories and accession number(s) can be found below: Genbank with accessions BankIt2528919 Seq1 OL878355 BankIt2528919 Seq2 OL878356 BankIt2528919 Seq3 OL878357 BankIt2528919 Seq4 OL878358 BankIt2528919 Seq5 OL878359 BankIt2528919 Seq6 OL878360 BankIt2528919 Seq7 OL878361 BankIt2528919 Seq8 OL878362 BankIt2528919 Seq9 OL878363 BankIt2528919 Seq10 OL878364.

## Ethics Statement

This study was approved by the institutional review board of Tongji hospital. Written informed consent was obtained from all participants. Experiments were conducted according to the Declaration of Helsinki.

## Author Contributions

SH participated in the research design, carried out the epidemiological investigation, undertook sequencing and genotyping, performed statistical analyses, and drafted the manuscript. DH collected samples, constructed vectors and executed the cell experiment. HW, S-YL, and C-ZL participated in the epidemiological investigation and collected samples for this study. DWeW participated in the research design, carried out the epidemiological investigation. GC conceived the study, participated in the research design, collected samples, and edited the final manuscript. JJ carried out the epidemiological investigation and collected samples. DWuW provided intellectual support for the revised manuscript. All authors have read and approved the final manuscript.

## Funding

This work was supported by the National Natural Science Foundation of China (Nos. 81770351 and 81630010) and the National Precision Medicine Program (No. SQ2017YFSF090157).

## Conflict of Interest

The authors declare that the research was conducted in the absence of any commercial or financial relationships that could be construed as a potential conflict of interest.

## Publisher's Note

All claims expressed in this article are solely those of the authors and do not necessarily represent those of their affiliated organizations, or those of the publisher, the editors and the reviewers. Any product that may be evaluated in this article, or claim that may be made by its manufacturer, is not guaranteed or endorsed by the publisher.

## Abbreviations

CHD: coronary heart disease
FOXA1: forkhead box A1
GWAS: genome-wide association study
HDL-C: high-density lipoprotein cholesterol
LD: linkage disequilibrium
LDL-C: low-density lipoprotein cholesterol
*SCARB1*: Scavenger receptor Class B Type 1
SNP: single nucleotide polymorphism.
