# Supplementary material for: Functional Deletion/Insertion Promoter Variants in SCARB1 Associated With Increased Susceptibility to Lipid Profile Abnormalities and Coronary Heart Disease
Source: Front Cardiovasc Med. 2022 Jan 13;8:800873. doi: 10.3389/fcvm.2021.800873 (PMC8793335; doi:10.3389/fcvm.2021.800873)
Supplement: Supplementary file 1 [file Table_1.DOC]

**Supplementary Figures**

(a)


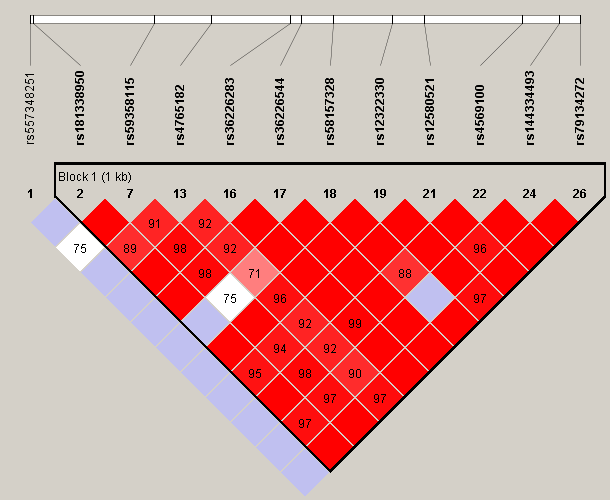
(b)

**Supplementary Figure**
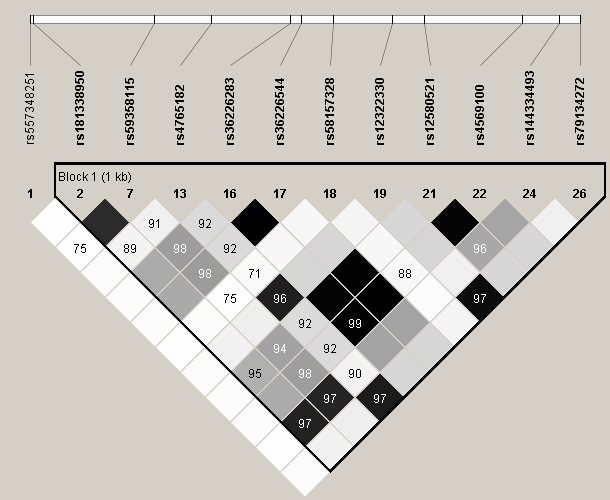
**1. LD structure and Haplotype Blocks of the SCARB1 promoter region.** LD (D’) (1a) and r^2(1b) for identified polymorphisms in

SCARB1 promoter region, as generated by Haploview 4.0 from the genotype

data of the 400 controls subjects. Haplotype blocks derived from these genotypes using the solid spine LD setting are outlined in black.


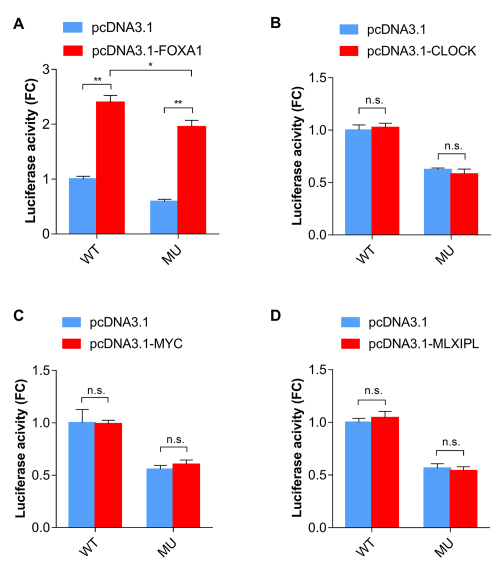


**Supplementary Figure 2. Interaction between transcription factors and variant rs144334493 in HepG2 cells.** HepG2 cells were co-transfected with pcDNA3.1(+) empty vector or pcDNA3.1(+)-FOXA1 construct (A), pcDNA3.1(+)-CLOCK construct (B), pcDNA3.1(+)-MYC construct (C), pcDNA3.1(+)-MLXIPL construct (D), and rs144334493 luciferase constructs using Lipo2000 (Lipofectamine 2000 [Invitrogen, Carlsbad, California]), according to the manufacturer’s instruction. Luciferase activity is expressed as fold change relative to the pcDNA3.1(+) empty vector and normalized by Renilla luciferase activity for each sample. Values are mean ± SE of three independent experiments each corresponding to at least six replicates. FC, fold change; **, p<0.01; *, p<0.05; n.s., no significant; WT, rs144334493 wide type construct; MU, rs144334493 mutation type construct.


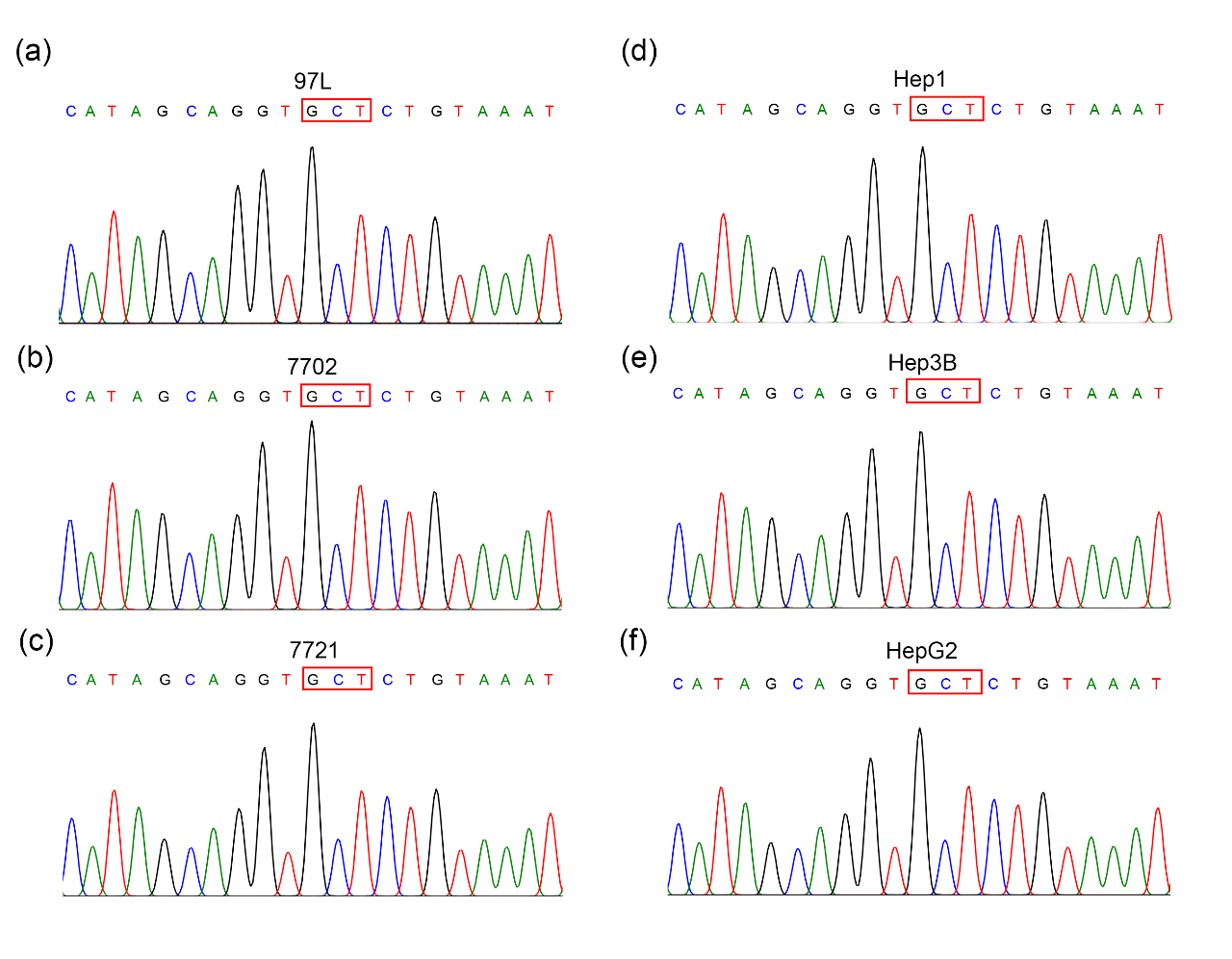


**Supplementary Figure 3. DNA sequences for 6 different hepatic cell lines.** All were homozygote major genotype of rs144334493 (indicated in red rectangle).


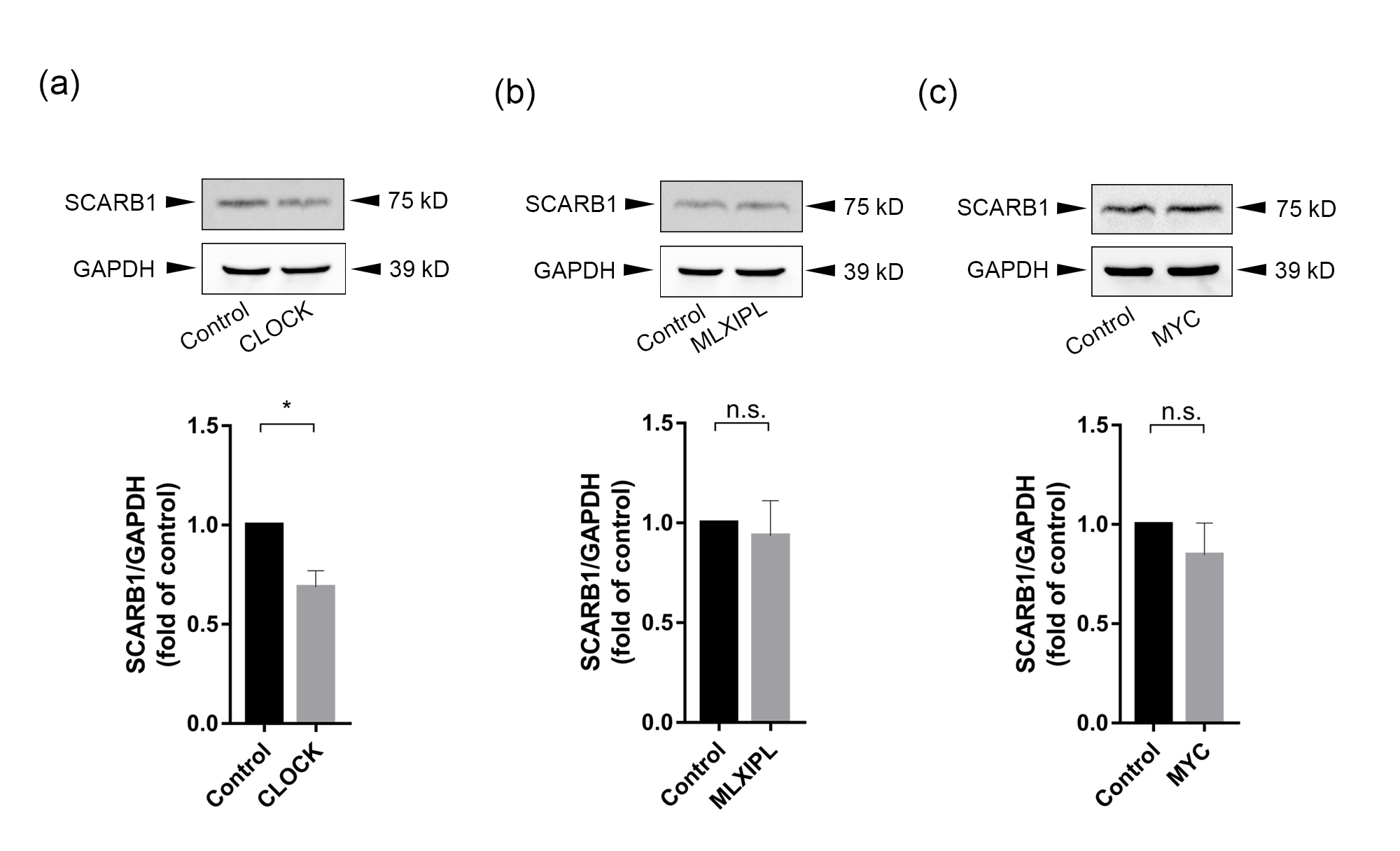


**Supplementary Figure 4. Expression studies of SCARB1 in HepG2 cells.** SCARB1 protein level is expressed as fold change of transcription factor(a, b, c, indicate CLOCK, MLXIPL, MYC construct, respectively) construct intervention relative to the pcDNA3.1(+) empty vector transfection. Values are mean ± SD of three independent experiments. *, p<0.05; n.s., no significant.

**
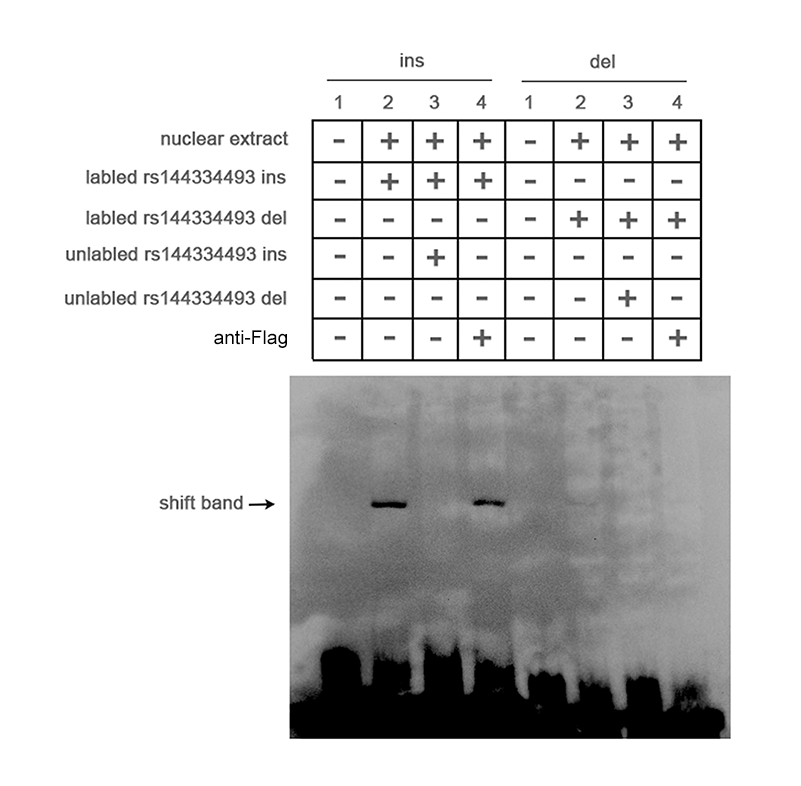
**

**Supplementary Figure 5. In vitro FOXA1 binding to rs144334493 insertion allele.** EMSA was performed with biotin-labeled probe containing either rs144334493 insertion allele or rs144334493 deletion allele (lane 1), with nuclear extract from HepG2 cells without or with unlabeled competing oligonucleotides (lane 2 and lane 3, respectively) or with anti-Flag antibody as indicated (lane 4). EMSA = Electrophoretic mobility gel shift assay. ins = rs144334493 insertion allele probe, del = rs144334493 deletion allele probe.
