# Supplementary material for: Functional Deletion/Insertion Promoter Variants in SCARB1 Associated With Increased Susceptibility to Lipid Profile Abnormalities and Coronary Heart Disease
Source: Front Cardiovasc Med. 2022 Jan 13;8:800873. doi: 10.3389/fcvm.2021.800873 (PMC8793335; doi:10.3389/fcvm.2021.800873)
Supplement: Supplementary file 2 [file Table_2.DOC]

**Supplementary Tables**

| **Supplementary Table 1:** Primers used for PCR and sequencing | | | |
| --- | --- | --- | --- |
| **Gene Name** | **Primer Coverage** | **Forward Primer Sequence** | **Reverse Primer Sequence** |
| SCARB1 | Promoter-1 | GCCTGGATTTCTCTGAACCTGT | GATCACCTGAGGTCGGGAGTT |
|  | Promoter-2 | GAGATGGAGTTTCGCTCTTGTT | CAGGTGGCCAGTGGTTTTAT |

| **Supplementary Table 2:** Primers used for reporter vector construciton | | | |
| --- | --- | --- | --- |
| **Gene Name** | **Primer Coverage** | **Forward Primer Sequence** | **Reverse Primer Sequence** |
| SCARB1 | rs144334493 | 5’ CGACGCGTTCTCACCTCAGCCACTCTGACC 3’ | 5’ CCCAAGCTTGCAGGCTCAGTCCTTGAGTGC 3’ |
|  | rs79134272 | 5’ CGACGCGTCTTGCCCCAGGACTCCAAT 3’ | 5’ CCCAAGCTTGCAGGGCCATCCTTAAGCA 3’ |
|  | rs4765182 | 5’ CGACGCGTGCCATTGTGTGCAAAGCACT 3’ | 5’ CCCAAGCTTTAACGCTGGTGCCACCATC 3’ |

**Supplementary Table 3: Primers used for transcription factor vectors construction**

| **Gene name** | **Gene ID** | **Forward Primer** | | **Reverse Primer** |
| --- | --- | --- | --- | --- |
| CLOCK | NM_001267843.1 | 5' CTAGCGTTTAAACTTAAGCTTATG  TTGTTTACCGTAAGCTGTAGTAAA 3' | 5’ TGCTGGATATCTGCAGAATTCCTACTGTGGTTGAACCTTGGAAG 3’ | |
| MYC | NM_001354870.1 | 5' CCCAAGCTTATGGATTTTTTTCGGGTAGTGG 3' | | 5' CGGAATTCTTACGCACAAGAGTTCCGTAGC 3' |
| MLXIPL | NM_032951.2 | 5’ CCCAAGCTTATGGCCGGCGC  GCTGGCAGGTCTGGCCGCG 3’ | | 5’ GGGGTACCCTATAAAGGTTTGCCAAGGGTGCCCTCTG 3’ |
| FOXA1 | NM_004496.3 | 5' CCCAAGCTTATGTTAGGAACTGTGAAGATGGAAG 3' | | 5' CGGAATTCCTAGGAAGTGTTTAGGACGGGTC 3' |

| **Supplementary Table 4:** Sequence of probes and primers sets | | | |
| --- | --- | --- | --- |
| **Rs ID** | **primer(5'→3')** | **probe(5'→3')** | **allele** |
| rs144334493 | F 5' GTGCCAGGCAAAGCTGTCT 3' | 5' FAM-AACATAGCAGGTGCTCTGT-MGB 3' | GCT |
|  | R 5' CCTTAAGCAACATGTCTTCGCCT 3' | 5' VIC-AGAACATAGCAGGTCTGTAAA-MGB 3' | - |
| rs557348251 | 5' FAM-CAGGCCCCATAGACGTTTTG 3' | 5' CAGGTGGCCAGTGGTTTTATG 3' |  |

FAM, 6-carboxyfluorescein; HEX, hexachloro-6-carboxyfluorescein. MGB indicates MGB probe.

| **Supplementary Table 5:** Primers used for CHIP assay | | | |
| --- | --- | --- | --- |
| **Gene Name** | **Primer Coverage** | **Forward Primer Sequence** | **Reverse Primer Sequence** |
| SCARB1 | rs144334493 | AGTCTCACCTCAGCCACTCTGAC | AACTCCAGAAGAAAGCAGCCTT |
| control | rs2070600 | GAAGCTTGGAAGGTCCTGTCT | GGAAAATCCCCTCATCCTGGAT |

| **Supplementary Table 6:** Probes used for EMSA assay | | |
| --- | --- | --- |
| **Primer Coverage** | **Forward Probe Sequence** | **Reverse Probe Sequence** |
| Foxa1--consensus | 5' TGTTTACTTTGTGTTTGTACA 3' | 5' TGTACAAACACAAAGTAAACA 3' |
| rs144334493-GCT | 5' CATAGCAGGTGCTCTGTAAATAT 3' | 5' ATATTTACAGAGCACCTGCTATG 3' |
| rs144334493-del | 5' ATATTTACAGACCTGCTATG 3' | 5' CATAGCAGGTCTGTAAATAT 3' |

| **Supplementary Table 7:** Baseline characteristics of the real-time RT-PCR samples | |
| --- | --- |
| **Characteristics** | **Controls (n=64)** |
| Age, yrs | 59.7±9.1 |
| Men, % | 62.7 |
| SBP, mm Hg | 126.7±17.8 |
| DBP, mm Hg | 79.9±12.3 |
| Hypertension, % | 0 |
| Diabetes, % | 0 |
| Hyperlipidemia, % | 0 |
| Smokers, % | 0 |
| TC(mmol/L) | 3.87±0.70 |
| HDL(mmol/L) | 1.10±0.30 |
| LDL(mmol/L) | 2.28±0.63 |
| TG (mmol/L) | 1.41±0.63 |
| SBP, systolic blood pressure; DBP, diastolic blood pressure; |  |
| Values are expressed as mean ± SD ; HDL-C = high-density lipoprotein cholesterol; LDL-C = low-density lipoprotein cholesterol; TC = total cholesterol; TG = triglycerides. | |

| **Supplementary Table 8.** Primers used for quantitative real-time PCR | | | |
| --- | --- | --- | --- |
| **Gene Name** | **Gene ID** | **Forward Primer Sequence** | **Reverse Primer Sequence** |
| SCARB1 | 949 | AATAAGCCCATGACCCTGAAG | TCGGAATGCCAGAAGTCAAC |
| ACTB | 60 | AGGGAAATCGTGCGTGACAT | CGTTGCCAATAGTGATGACC |

| **Supplementary Table 9. Association of rs144334493 genotype with plasma lipid levels** | | | | | | | | |
| --- | --- | --- | --- | --- | --- | --- | --- | --- |
|  | **Rs144334493** | | |  |  |  |  |  |
| **The general population** | **Homozygote major (N)** | **Heterozygote(N)** | **Homozygote minor (N)** | **Beta（SE）** | **Trend* p Value** | **Beta（SE）** | **Variance Explained (%)** | **Trend^†^ p Value** |
| TG | 3948 | 1140 | 87 | -0.060 (0.094) | 0.521 | -0.066 (0.133) | 0.0 | 0.621 |
| HDL | 3948 | 1140 | 87 | -0.002 (0.036) | 0.946 | 0.091 (0.089) | 0.2 | 0.308 |
| LDL | 3948 | 1140 | 87 | -0.104 (0.084) | 0.219 | -0.103 (0.207) | 0.0 | 0.621 |
| TC | 3948 | 1140 | 87 | -0.117 (0.104) | 0.264 | -0.039 (0.194) | 0.0 | 0.839 |
| APOA1 | 3948 | 1140 | 87 | -0.193 (0.083) | 0.021 | -0.188 (0.083) | 1.2 | 0.024 |
| APOB | 3948 | 1140 | 87 | 0.050 (0.081) | 0.539 | 0.048 (0.082) | 0.1 | 0.557 |

* Analysis was adjusted for sex and age. **^†^** Analysis was adjusted for sex, age, smoking, hypertension, and diabetes mellitus, and rs557348251.

| **Supplementary Table 10. Association of rs557348251 genotype with plasma lipid levels** | | | | | | | | |
| --- | --- | --- | --- | --- | --- | --- | --- | --- |
|  | **Rs557348251** | | |  |  |  |  |  |
| **The general population** | **Homozygote major (N)** | **Heterozygote (N)** | **Homozygote minor (N)** | **Beta（SE）** | **Trend* p Value** | **Beta（SE）** | **Variance Explained (%)** | **Trend^†^ p Value** |
| TG | 4933 | 234 | 8 | 0.055 (0.058) | 0.339 | -0.140 (0.094) | 0.4 | 0.135 |
| HDL | 4933 | 234 | 8 | 0.026 (0.022) | 0.232 | 0.012 (0.060) | 0.0 | 0.845 |
| LDL | 4933 | 234 | 8 | 0.034 (0.051) | 0.498 | 0.293 (0.124) | 0.8 | 0.019 |
| TC | 4933 | 234 | 8 | 0.050 (0.064) | 0.436 | 0.270 (0.131) | 0.5 | 0.040 |
| APOA1 | 4933 | 234 | 8 | -0.084 (0.049) | 0.089 | -0.086 (0.049) | 0.6 | 0.080 |
| APOB | 4933 | 234 | 8 | -0.004 (0.048) | 0.933 | -0.013 (0.048) | 0.0 | 0.787 |

* Analysis was adjusted for sex and age. **^†^** Analysis was adjusted for sex, age, smoking, hypertension, and diabetes mellitus, and rs144334493.

| **Supplementary Table 11:** Angiographic characteristics of rs144334493 patients | | | | |
| --- | --- | --- | --- | --- |
|  | **rs144334493** | | |  |
| **Variable** | **Ins/ins (3784)** | **Ins/del (1124)** | **Del/del (94)** | ***p value** |
| Coronary artery disease,% |  |  |  |  |
| LAD | 3366 (91.0) | 981 (89.6) | 83 (94.3) | 0.747 |
| LCX | 2126 (53.0) | 650 (54.9) | 58 (52.9) | 0.240 |
| RCA | 2267 (57.5) | 716 (60.5) | 60 (62.9) | 0.005 |
| Multivessel disease, % | 2610 (69.0) | 797 (70.9) | 64 (68.1) | 0.009 |

LAD = left anterior descending; LCX = left circumflex artery; RCA = right coronary artery; ins, insertion allele; del, deletion allele; *p value was abtained with multivariate unconditional logistic regression analysis by adjusting for sex, age, smoking, hypertension, hyperlipidemia and diabetes using dominant model.

| **Supplementary Table 12:** Angiographic characteristics of rs557348251 patients | | | | |
| --- | --- | --- | --- | --- |
|  | **rs557348251** | | |  |
| **Variable** | **Ins/ins (n = 4513)** | **Ins/del (n = 454)** | **Del/del (n = 35)** | **p value*** |
| Coronary artery disease,% |  |  |  |  |
| LAD | 3989 (88.4) | 409 (90.1) | 32 (91.4) | 0.934 |
| LCX | 2578 (57.1) | 234 (51.5) | 27 (77.1) | 0.078 |
| RCA | 2745 (60.8) | 271 (59.7) | 28 (80.0) | 0.065 |
| Multivessel disease, % | 3124 (69.2) | 316 (69.6) | 32 (91.4) | 0.043 |

LAD = left anterior descending; LCX = left circumflex artery; RCA = right coronary artery; ins, insertion allele; del, deletion allele; *p value was abtained with multivariate unconditional logistic regression analysis by adjusting for sex, age, smoking, hypertension, dyslipidemia and diabetes using resessive model.

| **Supplementary Table 13:** Linkage disequilibrium of SNPs in the promoter region of SCARB1 with known GWAS loci. | | | | | |
| --- | --- | --- | --- | --- | --- |
| **Known GWAS loci** | **Gene Position** | **SNP** | **Gene Position** | **D'** | **r2** |
| rs4765623 | 12:125320850 | rs79134272 | 12:125350530 | 0.592 | 0.204 |
| rs4765623 | 12:125320850 | rs12322330 | 12:125349849 | 0.592 | 0.204 |
| rs4765623 | 12:125320850 | rs4765182 | 12:125349194 | 0.609 | 0.207 |
| rs11057864 | 12:125335950 | rs79134272 | 12:125350530 | 0.668 | 0.013 |
| rs11057864 | 12:125335950 | rs12322330 | 12:125349849 | 0.668 | 0.013 |
| rs11057864 | 12:125335950 | rs4765182 | 12:125349194 | 0.696 | 0.014 |
| rs11057864 | 12:125335950 | rs59358115 | 12:125348988 | 1 | 0.013 |
| rs11057864 | 12:125335950 | rs181338950 | 12:125348548 | 1 | 0.013 |
| rs11057864 | 12:125335950 | rs4569100 | 12:125350320 | 0.733 | 0.091 |
| rs11057864 | 12:125335950 | rs12580521 | 12:125349968 | 0.73 | 0.089 |
| rs11057864 | 12:125335950 | rs36226544 | 12:125349519 | 0.735 | 0.092 |
| rs11057864 | 12:125335950 | rs36226283 | 12:125349480 | 0.735 | 0.092 |
